# Comparative analysis of salicylic acid levels and gene expression in resistant, tolerant, and susceptible cassava varieties following whitefly-mediated SLCMV infection

**DOI:** 10.1038/s41598-023-40874-3

**Published:** 2023-08-21

**Authors:** Srihunsa Malichan, Nattachai Vannatim, Somruthai Chaowongdee, Pornkanok Pongpamorn, Atchara Paemanee, Wanwisa Siriwan

**Affiliations:** 1 Department of Plant Pathology, Faculty of Agriculture, Kasetsart University, Bangkok, 10900 Thailand; 2 Center for Agricultural Biotechnology, Kasetsart University, Kamphaengsaen Campus, Nakhon Pathom, 73140 Thailand; 3 Center of Excellence on Agricultural Biotechnology (AG-BIO/MHESI), Bangkok, 10900 Thailand; 4 National Omics Center, National Science and Technology Development Agency (NSTDA), Pathum Thani, 12120 Thailand

**Keywords:** Biotechnology, Molecular biology, Plant sciences

## Abstract

*Sri Lankan cassava mosaic virus* (SLCMV), the primary pathogen responsible for cassava mosaic disease in cassava plantations, is transmitted via infected cutting stems and the whitefly vector, *Bemisia tabaci*. To obtain better insights into the defense mechanism of cassava against SLCMV, whiteflies were used to induce SLCMV infection for activating the salicylic acid (SA) signaling pathway, which triggers the innate immune system. The study aimed to investigate the specific interactions between viruliferous whiteflies and SA accumulation in resistant (C33), tolerant (Kasetsart 50; KU50), and susceptible (Rayong 11) cassava cultivars by infecting with SLCMV. Leaf samples were collected at various time points, from 1 to 7 days after inoculation (dai). The SA levels were quantified by gas chromatography–mass spectrometry and validated by quantitative reverse transcription polymerase chain reaction. The SA levels increased in KU50 and C33 plants at 2 and 3 dai, respectively, but remained undetected in Rayong11 plants. The expression of *PR-9e*, *PR-7f5*, *SPS1*, *SYP121*, *Hsf8*, and *HSP90* increased in infected C33 plants at 4 dai, whereas that of KU50 plants decreased immediately at 2 dai, and that of Rayong11 plants increased at 1 dai but gradually decreased thereafter. These findings strongly indicate that SA plays a crucial role in regulating antiviral defense mechanisms, especially in SLCMV-resistant plants. Altogether, the findings provide valuable insights into the mechanisms underlying the activation of SA-mediated anti-SLCMV defense pathways, and the resistance, tolerance, and susceptibility of cassava, which can aid future breeding programs aimed at enhancing SLCMV resistance.

## Introduction

Cassava (*Manihot esculenta* Crantz) is one of the most widely consumed root crops worldwide, and provides stable sustenance in the form of carbohydrates to more than 800 million people^1^. The utilization of cassava-based production systems in industrial applications has increased steadily during the previous decade^2^. The production of cassava crop in Southeast Asia has been constrained by several disease outbreaks since 2016. *Sri Lankan cassava mosaic virus* (SLCMV), which was responsible for causing cassava mosaic disease (CMD) in Southeast Asia, has now spread to the major areas of cassava crop production in Cambodia^3^, Vietnam^4^, Thailand^5^, and Southern Laos^6^. Outbreaks of CMD have been occurring in Thailand since 2018, and are rapidly spreading to cassava plantations across 27 provinces^7^. The Department of Agriculture Extension, Thailand reported in 2022 that CMD-affected areas cover approximately 40,000 hectares of land. A previous study reported that CMD caused a maximum yield loss of 33% and reduced the starch content of numerous commercially important local cassava cultivars by 28%^4^. The Department of Agriculture (DOA) in Thailand has published a list of SLCMV-susceptible cultivars of cassava, including Rayong11, and has urged farmers to avoid planting these varieties. The DOA additionally promoted the cultivation of the Kasetsart 50 (KU50), Huay Bong 60, and Rayong 72 cultivars of cassava, which are tolerant to certain genotypes of SLCMV. The breeding program of Kasetsart University has introduced the C33 cultivar of cassava from the International Center for Tropical Agriculture (CIAT) as germplasm for developing CMD-resistant cultivars to check SLCMV infections and enhance the production of cassava. The C33 cultivar contains the single dominant *CMD2* locus, which confers resistance to CMD^8^.

SLCMV is a member of the *Begomovirus* genus under the *Geminiviridae* family, and its genome comprises two single-stranded DNA molecules (DNA-A and DNA-B), each of ~ 2.8 Kb. The whitefly (*Bemisia tabaci*) is the only know insect vector of CMD. In Thailand, SLCMV is specifically transmitted by the Asia II 1 species of the *B*. *tabaci* cryptic species complex^7^. An earlier study by Delaquis*, *et al*.*^9^ identified that the mobilization of infected planting material, namely, stem cuttings, was the major source of inoculum for the spread of CMD in Southeast Asia. Additionally, the outbreaks of whitefly in planting areas can result in a wider spread of CMD and significant yield losses of cassava^10,11^.

Whitefly vectors, predominantly including *B*. *tabaci*, pose as a considerable economic challenge to the growth of cassava crop. These insects are phloem feeders and cause minimal damage to plant cells during feeding, and thereby differ from aphids that puncture cells during probing^12^. It has been reported that the enzymes and effector molecules secreted by whiteflies during the process of feeding trigger the defense mechanisms of plants^13,14^, thereby supporting the complex interactions between plants and phloem feeders that originate in the phloem. As several phloem feeders transmit plant pathogens, plants have developed intricate defense mechanisms in response to insect infestation, which are comparable to the mechanisms activated during plant-pathogen interactions^15,16^. Recent studies have demonstrated that the strong defense reaction of plants against whitefly-transmitted viral diseases is triggered by the accumulation of free salicylic acid (SA)^17^.

SA is the key signaling molecule necessary for activating the innate immune responses of plants against abiotic and biotic stresses. Stress-induced signaling and the accumulation of SA trigger substantial transcriptional reprogramming of defense-related genes and enhance the formation of secondary metabolites and antimicrobial chemicals, which ensure plant development and immunity^18^. SA-induced defense mechanisms play a significant role in regulating the basal and gene-for-gene defense response pathways against biotic pathogens^19^. SA signaling is activated by PAMP-triggered immunity (PTI) or effector-triggered immunity (ETI)^20^, depending on the nature of the abiotic or biotic stress perceived by the innate immune system of plants^21^. PTI is a conserved molecular mechanism that is specifically activated in response to plant pathogens identified by pattern recognition receptors (PRRs), and primarily initiates the innate immune response. ETI is triggered by effector resistance proteins, also known as nucleotide-binding leucine-rich repeats (NB-LRRs), and initiates a defense response in plants^22^.

SA upregulates the expression of plant defense genes that trigger the production and accumulation of antioxidant enzymes and steroid glycoalkaloids and induces the secretion of organic volatile compounds that attract the natural enemies of insect herbivores^23^. Additionally, the SA-dependent pathway confers systemic acquired resistance (SAR), a long-acting mechanism that is induced against a range of invasive pathogens, and is a mode of broad-spectrum resistance in plants^24^. It has been reported that the stimulation of the SA pathway in *Arabidopsis thaliana* is essential for basal and resistance gene (*R*)-mediated biotrophic pathogen defense^25^. Irigoyen et al.^26^ further reported that SA and jasmonic acid (JA) coordinately regulate the expression of the pathogenesis-related (PR) protein in response to whitefly infestation.

PR protein families include glucanases, chitinases, peroxidases, thaumatin-like proteins, and proteases that have a wide range of biological activities. *PR* genes are known to respond to multiple biotic stresses and defense hormones^27,28^. A recent study identified that *PR2*, *PR5*, *PR7*, and *PR9* are the key *PR* genes of cassava that respond to the whitefly-induced production of SA, JA, and various other biotic stress factors^26^.

The present study aimed to determine the precise role of the whitefly *B*. *tabaci* in SLCMV-whitefly interactions, especially in terms of SA production and accumulation in resistant, tolerant, and susceptible cultivars of cassava. Understanding the regulation of SA signaling pathways by whitefly vectors should provide valuable insights into the underlying plant defense mechanisms and facilitate the generation of SLCMV-tolerant/resistant genotypes of cassava for breeding purposes.

## Methods

### Generation of virus-free cassava plants

The stem tissues of three virus-free cassava stem genotypes (C33, Kasetsart 50, and Rayong 11) were provided by the Thai Tapioca Development Institute (TTDI), Nakhon Ratchasima, Thailand. All the cassava genotypes were grown in an insect-proof cage in the greenhouse of the Department of Plant Pathology (Kasetsart University, Bangkok, Thailand). One-month-old cassava plants were visually inspected for symptoms of SLCMV infection followed by indexing with polymerase chain reaction (PCR) using primers specific for the *AV1* gene of SLCMV^7^. DNA was extracted from 10 mg of fresh cassava leaves using the cetyltrimethylammonium bromide (CTAB) method^29^. PCR amplification was performed in a total reaction volume of 25 µL, containing 10 ng/µL DNA template, 12.5 µL of 2 × PCRBIO Taq DNA polymerase (PCR Biosystems Ltd., Pennsylvania, USA), and 0.4 µM of each of the forward (5′-GTTGAAGGTACTTATTCCC′-3) and reverse (5′-TATTAATACGGTTGTAAACGC′-3) primer pairs. The thermal cycling program was as follows: pre-denaturation for 5 min at 94 °C, followed by 35 cycles of denaturation for 1 min at 94 °C, annealing for 1 min at 58 °C, extension for 1 min at 72 °C, and a final extension for 5 min at 72 °C. The amplified products were examined for the presence of SLCMV, and positive and negative controls of SLCMV were included in the detection assays.

### Collection and rearing of *B*.* tabaci*

The pupal instar stage of *B*. *tabaci* whitefly larvae, free from the symptoms of CMD, were collected from cassava fields at Nakhon Ratchasima, Thailand. For the transmission of SLCMV, the pre-pupal instar stages of *B*. *tabaci* were reared on the Chao phaya cultivar of eggplant. Non-viruliferous whitefly colonies were obtained by transferring the newly enclosed adults for several generations on eggplant. The virus-free colonies of whitefly were grown on the host plants in an insect-proof cage and maintained by transferring to virus-free plants every 4–6 weeks. The whiteflies were reared at a temperature of 26 ± 2 °C, relative humidity of 60%, and under a photoperiod of 14 h/10 h in light/dark. The adult whiteflies were selected for the transmission of SLCMV.

### Transmission of SLCMV

Twenty non-viruliferous adult whiteflies were allocated per test plant. The whiteflies were exposed to a prestarvation period (PSP) of 30 min before acquisition, following which they were released into symptomatic cassava plants for 24 h. The viruliferous whiteflies were collected by aspiration and starved for 30 min, following which 20 whiteflies were released into the cages of virus-free cassava plants of three genotypes. A plastic clip apparatus was used during the 48 h inoculation access period^30^ and removed after 48 h. The plants were maintained at a temperature of 26 ± 2 °C and a relative humidity of 60% during the transmission of SLCMV.

### Sample collection

The third leaf of the uppermost shoot of all the three cassava genotypes (C33, Kasetsart 50, and Rayong 11) was collected at 1, 2, 3, 4, 5, 6, and 7 days after inoculation (dai). Sample collection was performed in triplicate at each time-point for all the three genotypes. The individual leaf samples were weighed and immediately stored at − 20 °C for further analysis.

### Sample preparation and gas chromatography-mass spectrometry (GC–MS) detection

Extraction was performed in this study as previously described, with modifications^31^. Briefly, 100 mg of fresh cassava leaves from each sample was used for extracting JA, abscisic acid (ABA), and SA. The cassava leaf tissues were ground using a glass rod and extracted with 750 μL of solvent comprising 75% methanol, 20% water, and 5% formic acid. The mixture was vortexed for 30 s and incubated on ice for 10 min. The cold mixture was next centrifuged at 121 × *g* for 5 min, following which the supernatant was collected and the leaf precipitates were re-extracted twice with the extraction solvent. The supernatant fractions were combined and dried using a centrifugal concentrator. The dried crude extracts were dissolved in 2 mL of 1 M formic acid and loaded onto OASIS HLB cartridges (1 cc, 30 mg; Waters, Milford, MA, USA). The cartridges were washed with 2 mL of 1 M formic acid and eluted with 2 mL of MeOH + 1% formic acid. The eluates were dried and derivatized as previously described^32^ before GC–MS analyzes.

The GCMS–QP2020 NX apparatus (Shimadzu Co., Japan) equipped with an SH-Rxi-5Sil-MS column (0.25 µm df × 0.25 mm ID × 30 m length) was used for GC–MS analysis in this study. Helium (99.9%) was used as the carrier gas, and the flow rate was adjusted to 1 ml/min. A 2 µL volume of sample was injected using the split mode with a split ratio of 1:10. The ion source (electron ionization (EI) at 70 eV) and interface temperatures of the mass spectrometer were maintained at 250 °C, and the temperature of the injector was maintained at 200 °C. The mass spectrometer was operated using both the scan (*m/z* 45–500, scan speed of 1666 and event time of 0.30 s) and SIM (*m/z* 267 → 268 & 209) acquisition modes. The oven temperature program of the column was as follows: initial temperature was set to 80 °C (held for 6 min), increased at a rate of 6 °C/min to 180 °C, and finally increased at a rate of 8 °C/min to 270 °C (held for 9 min). Standard SA (ChemFaces, CFN93274) was used for identification.

### Gene expression analysis

The total RNA was extracted using QIAzol Lysis Reagent (Qiagen, Hilden, Germany), according to the manufacturer’s protocol. The first-stand cDNA was synthesized using a RevertAid First Stand cDNA Synthesis Kit (ThermoFisher Scientific) and Oligo(dT)_18_. Quantitative reverse transcription PCR (RT-qPCR) was performed using the Hot FIREPol^®^ EvaGreen^®^ qPCR Mix Plus (Solis BioDyne, Tartu, Estonia), and the relative gene expression levels were determined using the ΔΔ*C*_t_ method. Ubiquitin 10 (*UBQ10*) was used as the reference gene as previously described^33^. The primers used for RT-qPCR in this study are enlisted in Table S1.

### Statistical analyses

The concentration of SA and the gene expression levels of *PR9e*, *PR7f5*, *HSP90*, *Hsf8*, *SPS1*, and *SYP121* obtained from three replicates per group were calculated using the Pearson correlation analysis model. One-way analysis of variance^34^ and Bonferroni test were used for comparing the treatments. The differences in the concentrations of SA were considered to be statistically significant at *p* < 0.01, and the analyzes were performed using the SAS statistical analysis package (SAS Institute, Cary, NC, USA).

## Results

### Phytohormone response of cassava plants to SLCMV infection

In this study, the levels of the phytohormones, including JA, ABA, and SA, in the infected leaves of the C33, KU50, and Rayong11 cassava varieties, which were susceptible, tolerant, and resistant to SLCMV, respectively, were evaluated following SLCMV infection. GC–MS analyses were performed for detecting the levels of phytohormones in the leaves. The results indicated that SA was the only phytohormone in all the samples. However, the levels of JA and ABA did not alter significantly following the inoculation of SLCMV via *B*. *tabaci* infestation in any of the three cassava genotypes studied herein. Altogether, the findings indicated that infection with SLCMV due to whitefly infestation induced the accumulation of SA, which was in turn affected by the genotype of cassava.

**Figure 1:**
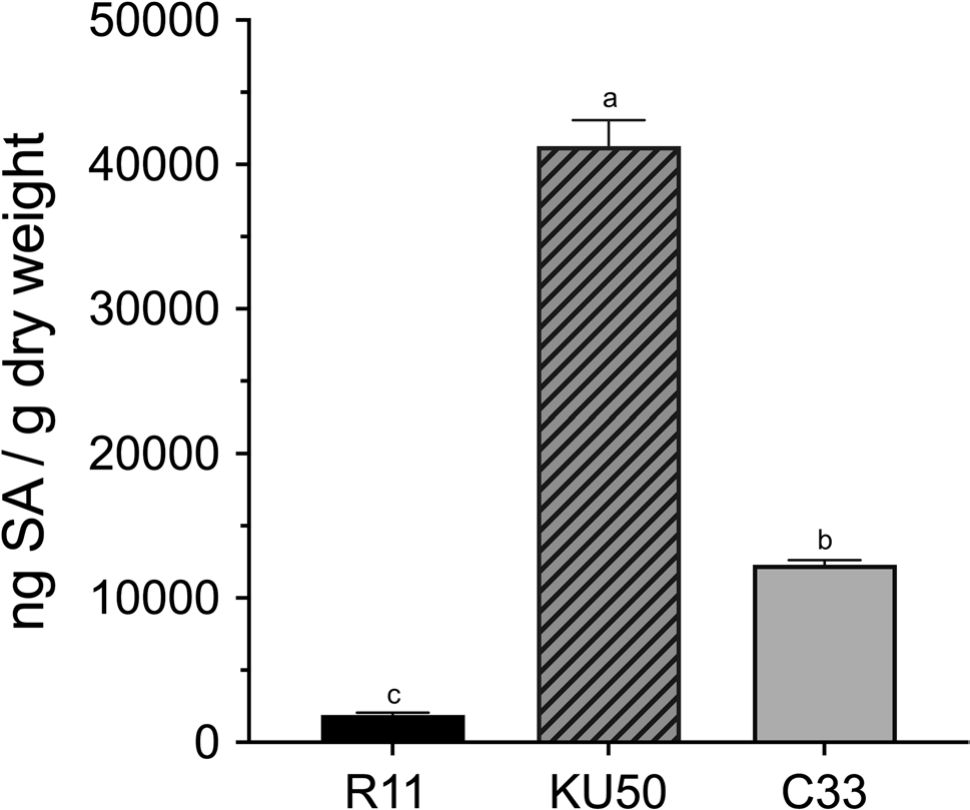
Comparison of the levels of SA in the leaves of the three different varieties of cassava infected with SLCMV (n = 3 per group).

The patterns of SA accumulation in the SLCMV-tolerant, -resistant, and -susceptible phenotypes of cassava were subsequently determined in this study. The results demonstrated that the level of SA in the KU50 cultivar reached approximately 41,000 ng/g, while those in the C33 and Rayong11 varieties were 1,200 and 250 ng/g, respectively. Statistical analyzes revealed distinct SA accumulation patterns among the three varieties of cassava, being highest in the KU50 cultivar and lowest in the Rayong11 variety. The differences in the levels of SA among the C33, KU50, and Rayong 11 varieties were found to be statistically significant at *p* < 0.01 (Fig. 1).

### SA levels in cassava leaves following SLCMV infection via *B*.* tabaci* infestation

The changes in the levels of SA in the leaves of the C33, KU50, and Rayong11 cassava genotypes in response to SLCMV infection were subsequently measured. SLCMV infection was initiated by whitefly infestation, and the leaves were collected at 1, 2, 3, 4, 5, 6, and 7 dai for analyzing the levels of SA. The time of induction of SA in the different phenotypes of cassava was determined following SLCMV infection via *B*. *tabaci* for elucidating the early response of SA induction in cassava plants to SLCMV infection.

**Figure 2:**
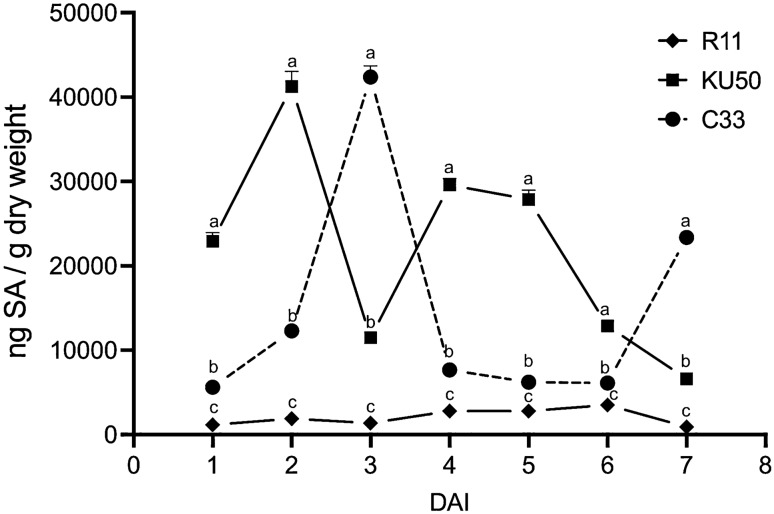
Levels of SA in the leaves of the three different varieties of cassava collected at 1, 2, 3, 4, 5, 6, and 7 dai (n = 3 per group).

The results demonstrated that SA was detected in the initial phase of infection in the KU50 and C33 cassava varieties following inoculation, which exhibited tolerance and resistance to SLCMV, respectively, following whitefly infestation. However, the production of SA was not observed in the susceptible phenotype following whitefly infestation. The results of statistical analyzes revealed distinct SA accumulation patterns among the three cassava varieties on the first day following inoculation. The level of SA was highest in the KU50 cultivar, and lowest in the Rayong11 variety. The differences in the levels of SA among the C33, KU50, and Rayong 11 varieties were statistically significant at *p* < 0.01 (Fig. 2).

The results demonstrated that that the concentration of SA was highest in the KU50 and C33 varieties at 2 and 3 dai, respectively. These findings suggested that the tolerant varieties of cassava have a more rapid response to both SLCMV infection and whitefly infestation, as indicated by the elevated levels of SA during the early stages of infection. In contrast, the susceptible Rayong11 phenotype did not exhibit a significant response in terms of SA production.

The pattern of SA accumulation in the KU50 and C33 varieties were slightly similar, which was characterized by a rapid increase in the production of SA immediately after inoculation, followed by a decrease in the SA levels on the subsequent day. Although the concentration of SA increased thereafter, it did not reach the same levels as those observed on the first day of inoculation. In contrast, the pattern of SA accumulation in the SLCMV-resistant variety of cassava indicated a delayed response compared to that of the tolerant variety of cassava.

Interestingly, SA was not detected in the susceptible Rayong11 variety of cassava at any point during experimentation. This finding suggested that SA is not induced in the susceptible variety of cassava following the inoculation of SLCMV via whitefly infestation, indicating that the plant defense system may not be fully activated in susceptible plants, which leads to the manifestation of severe disease symptoms.

### Validating gene response to infestation with viruliferous whiteflies

**Figure 3:**
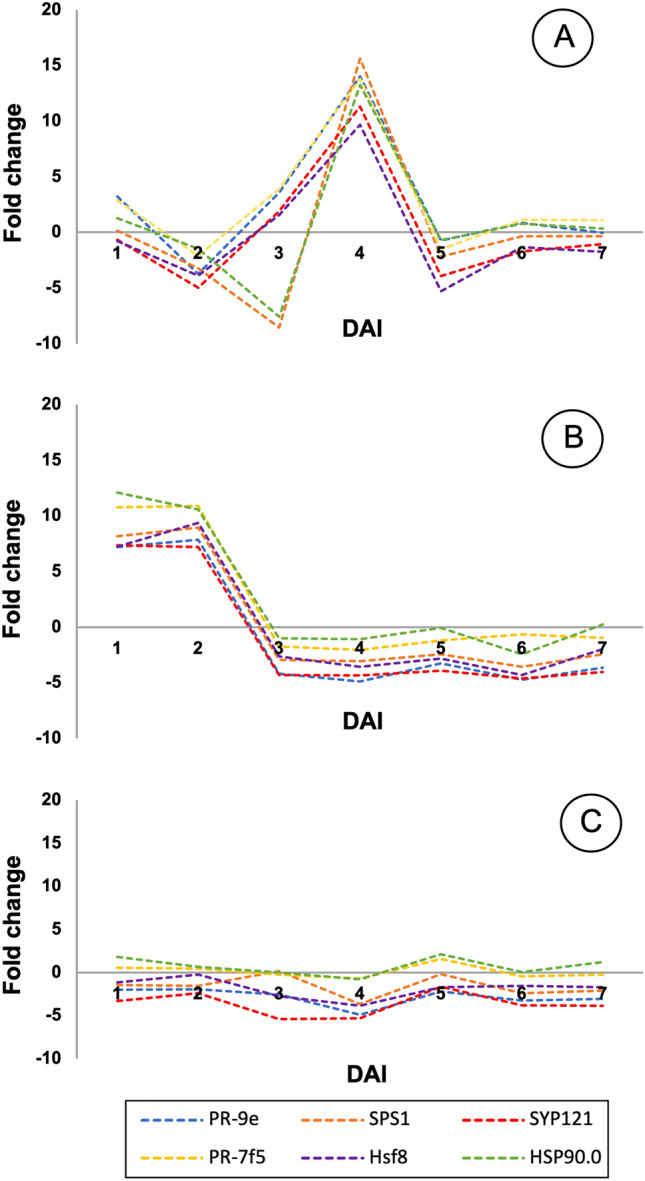
Analysis of the expression of genes related to the accumulation of SA. The *PR-9e*, *PR-7f5*, *SPS1*, *SYP121*, *Hsf8*, and *HSP90* genes were detected in the C33, KU50, and Rayong11 genotypes. The SLCMV-infected leaves inoculated by whitefly infestation were collected at 1, 2, 3, 4, 5, 6, and 7 dai (n = 3 per group).

In order to identify the genes that were involved in regulating the accumulation of SA, we selected *PR9e*, *PR7f5*, *HSP90*, *Hsf8*, *SPS1*, and *SYP121* as candidate genes^26,35,36^. The differentially expressed genes acted as regulators of SA in the three genotypes of cassava. The findings revealed that all the genes of interest were upregulated at 4 dai, and their expression reduced at 5 dai in the resistant C33 genotype. However, the expression of these genes was upregulated at 2 dai in the susceptible Rayong11 genotype, and subsequently downregulated before 3 dai until the final observation time-point. Notably, the expression of the candidate genes selected herein was downregulated in the KU50 variety (Fig. 3).

The concentration of SA and the expression values of *PR9e*, *PR7f5*, *HSP90*, *Hsf8*, *SPS1*, and *SYP121* were additionally calculated based on the triplicate data from each group using the Pearson correlation analysis model. The results of statistical analyses revealed that there was a strong association between the accumulation of SA and the expression of the *PR7f.* gene, with a correlation coefficient (r) of − 0.65542. However, the association between the accumulation of SA and the expression of the other genes were negligible or weak, with correlation coefficients of − 0.02044 and 0.10822, respectively.

### SA-dependent plant defense in resistant, tolerant, and susceptible phenotypes of cassava

The present study revealed that the accumulation of SA plays a crucial role in activating the *PR* genes during plant defense. The expression of *PR9e*, *PR7f5*, *HSP90*, *Hsf8*, *SPS1*, and *SYP121*, which induce SAR, was determined in this study, and the findings indicated that the genes are activated by the accumulation of SA. However, the responses of the three cassava phenotypes to SLCMV infection via whitefly vectors were distinct with respect to the SA-mediated plant defense systems.

The accumulation of SA was highest at 3 dai in the C33 variety, and the accumulated SA subsequently upregulated the six genes at 4 dai (Figs. 2 and 3A). This phenomenon was exclusively observed in the resistant phenotype. However, there was no significant increase in the concentration of SA in the Rayong11 variety following SLCMV infection caused by whitefly vectors, which downregulated the expression of the selected candidate genes (Figs. 2 and 3B). We also observed that the accumulation of SA in the KU50 cultivar was high at 2 dai, and the levels were comparable to those of the C33 variety. However, the results of gene expression analysis indicated that SA was unable to activate the *PR* genes in the KU50 cultivar (Figs. 2 and 3C).

## Discussion

CMD has emerged as a major challenge to the growth of cassava crop in recent years owing to the rapid transmission of SLCMV. Similar to CMD epidemics in Africa^37^, India^38^, and Vietnam^39^, the data obtained by our group on SLCMV infections in Thailand suggested that infected cassava cuttings serve as an initial source of SLCMV infection, whereas the whitefly *B*. *tabaci* is responsible for the secondary spread of the virus. The relationship between cassava mosaic virus and *B*. *tabaci* is complex relative to other types of virus-vector associations and requires further investigation.

The present study investigated the accumulation of SA following the infection of different cassava phenotypes with SLCMV via the whitefly vector, *B*. *tabaci*. The species of whitefly was confirmed prior to the inoculation of SLCMV by PCR amplification of the *mtCO1* gene^40^. The results of nucleotide sequencing confirmed the whitefly species to be *B*. *tabaci* Asia II 1 cryptic species.

The levels of SA accumulated in the leaves of the resistant (C33), tolerant (KU50), and susceptible (Rayong11) cultivars of cassava following SLCMV infection were quantified from the intercellular trafficking phase of viral infection to the stage of virulent infestation. It has been reported that SA has various effects on viral replication, long-distance movement, and the intercellular trafficking phases during the viral infection cycle in infected plants^41,42^. It has been additionally reported that SA plays an important role in regulating disease resistance^43–45^ and whitefly-mediated infection in cassava^26^.

In this study, we observed a transient increase in the levels of SA following the infestation of viruliferous *B*. *tabaci* in KU50 and C33 genotypes, and the levels of SA at 2 and 3 dai decreased at 3 and 4 dai, respectively. In contrast, the levels of SA remained low in the Rayong11 genotype throughout the duration of the experiment. This finding suggested that the inoculation of SLCMV via *B*. *tabaci* infestation led to the accumulation of SA in the different cassava cultivars. The results demonstrated that the initiation of SA accumulation varied among the genotypes, especially in the resistant genotypes, which indicated a genotype-specific response to *B*. *tabaci* infestation. A previous study demonstrated the accumulation of SA occurred transiently in response to *B*. *tabaci* infestation at 2 dai, but decreased at 12 dai in four genotypes of tomato^46^. Another study by Zhang and co-workers^47^ similarly demonstrated a transient increase in the levels of SA at 7 dai in lima bean plants.

SA is known to regulate extreme resistance (ER), indicated by the presence of necrotic lesions in plants carrying the *R* gene, and ER is conceptually similar to ETI^20^. This phenomenon has been documented in the resistance of tobacco plants to *Tomato Bushy Stunt Virus* (TBSV)^48^ and the resistance of soybean to *Soybean Mosaic Virus* (SMV)^49^ The data obtained in this study confirm that SA accumulates in both resistant and tolerant genotypes of cassava. The presence of high levels of SA can serve as a potential indicator of resistance or susceptibility to SLCMV. Furthermore, the elevated levels of SA appear to play a significant role in delaying the onset and progression of symptoms of CMD.

Interestingly, the results of RT-PCR revealed that the expression levels of the *PR* and stress-related genes increased following infection, especially in the resistant C33 variety at 4 dai (Figs. 2 and 3A). This finding is interesting because it indicates a delayed peak in gene expression following the highest accumulation of SA at 3 dai. This suggests that there may be a temporal delay or regulatory mechanism involved in the activation of *PRs* and SA-related genes following the accumulation of SA in the resistant variety. Further studies are necessary for elucidating the underlying mechanisms and determining their implications in plant defense responses against SLCMV.

The present study revealed that the expression of SA was highest in the tolerant KU50 genotype at 2 dai, and the production of SA in this cultivar was initiated before that of the other genotypes. Notably, the gene expression levels in the KU50 cultivar were high at 2 dai and decreased immediately thereafter from 3 to 7 dai. This transient expression pattern suggests that the induction of SA-related genes in the KU50 cultivar represents an early response to the pathogen, which was followed by a rapid downregulation of gene expression. This dynamic regulation of gene expression may indicate the activation of other defense mechanisms or the establishment of a state of resistance in the tolerant genotype. Further investigations are necessary for unraveling the underlying mechanisms governing this temporal gene expression pattern and for determining its functional significance in conferring tolerance to SLCMV.

The *PR* genes of cassava have been investigated in terms of response to infestation by whiteflies, other diseases, and insect carriers, including the cassava mealybug (*Phenacoccus manihoti*), bacterial blight disease (*Xanthomonas axonopodis* pv. *manihotis*), anthracnose disease (*Colletotrichum gloeosporioides*), CMD (*cassava mosaic virus*), and cassava brown streak disease (*cassava brown streak virus*)^50–55^. Irigoyen et al.^26^ characterized the *PR* genes of cassava that respond to the canonical defense hormone, SA. The *PR7* and *PR9* genes have been shown to play important roles in degrading the cell walls of pathogens and fortifying the host cell wall. The *PR9* gene also encodes lignin-forming peroxidases that reinforce the host cell wall by catalyzing lignification and preventing the penetration of pathogens^56,57^.

The present study revealed that the activation and expression of the *PR* genes, specifically *PR7* and *PR9*, were dependent on the accumulation of SA in the plant defense mechanism. This defense response is characterized by the accumulation of SA, which activates the *PR* genes that are involved in pathogen defense. The induction of *PR7* and *PR9* indicates the involvement of SA-mediated signaling pathways that are responsible for triggering an effective pathogen defense response. These findings highlight the significance of SA-dependent mechanisms in orchestrating plant defenses and provide valuable insights into the molecular basis of plant-pathogen interactions.

It has been reported that geminiviruses are capable of adapting to and counteracting the SA-mediated pathogen defense response, enabling them to evade SA-regulated antiviral defense mechanisms. Being highly specialized plant pathogens, geminiviruses have developed various strategies for interfering with SA-mediated signaling pathways that are crucial for plant defense against viral infections. Geminiviruses can effectively establish and propagate within host plants by evading or suppressing the SA response^58^. The downregulation of the *PR* genes in the SLCMV-susceptible (Rayong11) and SLCMV-tolerant (KU50) cultivars following whitefly infestation could be attributed to the effector molecules secreted by the whiteflies, which actively suppressed plant immunity in these genotypes^59^. Whitefly-mediated viral infections have been reported to promote rather than suppress SA-regulated defense responses and the expression of *PR* genes in *A*. sp.^60^. The present study indicated that the regulation of the expression of *PR* genes and patterns of SA accumulation in cassava may be distinct from other species and across different cultivars. Altogether, the results suggested that the susceptible Rayong11 and KU50 genotypes are unable to activate SLCMV resistance at the beginning of viral infection owing to the suppression of SA accumulation and expression of *PR* genes.

## Conclusions

In this study, three cassava cultivars, including the SLCMV-resistant C33, SLCMV-tolerant KU50, and SLCMV-susceptible Rayong11 cultivars, were inoculated with SLCMV via whitefly infestation and the SA contents were analyzed after 1, 2, 3, 4, 5, 6, and 7 dai. The findings revealed that the accumulation of SA was highest at 3 dai in the C33 genotype, and the expression of *PR* and SA-related genes subsequently increased at 4 dai. These results strongly indicate that SA plays a crucial role in regulating the antiviral defense mechanisms of cassava, especially in the resistant C33 genotype. The levels of SA peaked at 2 dai in the KU50 genotype, which was followed by the downregulation of gene expression. These findings suggested that the tolerant genotype may have defense mechanisms distinct from those of the other genotypes. The accumulation of SA and the gene expression levels decreased stably in the susceptible Rayong11 phenotype throughout the course of SLCMV inoculation until the final day of sample collection. These findings suggested that the accumulation of SA was impaired in the susceptible phenotype, indicating that the defense response against SLCMV was compromised in this cultivar. Contrary to previous findings, the results of the present study demonstrated the accumulation of SA in cassava following the inoculation of SLCMV, which induced SAR against SLCMV infection. The findings revealed that SA-induced SAR appeared to be more effective in the resistant variety. However, further studies are necessary for elucidating the specific interactions between SA and the SAR pathway, and for characterizing the SA-induced resistance network against SLCMV. An understanding of these underlying mechanisms would aid in developing effective strategies for managing SLCMV infections in cassava crop plantations.

### Supplementary Information

Supplementary Tables.

## Data Availability

The data sets supporting the conclusions of this article are included with the article and its additional files. Raw data of GC–MS analysis of cassava leaves are available at https://www.scidb.cn/s/aMV36r. All methods and plant material used in the study is in accordance with the relevant institutional, national, and international guidelines and legislation.
