# Supplementary material for: Comparative analysis of salicylic acid levels and gene expression in resistant, tolerant, and susceptible cassava varieties following whitefly-mediated SLCMV infection
Source: Sci Rep. 2023 Aug 21;13:13610. doi: 10.1038/s41598-023-40874-3 (PMC10442324; doi:10.1038/s41598-023-40874-3)
Supplement: Supplementary file 1 — Supplementary Tables. [file 41598_2023_40874_MOESM1_ESM.pdf]

Additional Data

Comparative analysis of salicylic acid levels and gene expression in resistant, tolerant, and susceptible cassava varieties following whitefly-mediated SLCMV infection

Srihunsa Malichan[1], Nattachai Vannatim[1], Somruthai Chaowongdee[2], Pornkanok Pongpamorn[3], Atchara Paemanee[3], and Wanwisa Siriwan[1*]

Table S1 Primer list

No	Gene Name	Primer Name	Primer Sequence (5' ⟶ 3')	References
1	*PR-7f5*	MePR-7f5F	CAGTCCCCAAGAAATGGAGA	Irigoyen et al (2020)
2	*PR-7f5*	MePR-7f5R	TCCTGAACGGTGTTTGATGA	Irigoyen et al (2020)
3	*PR-9e*	MePR-9eF	GGAGGCCATGCTTACTGATC	Irigoyen et al (2020)
4	*PR-9e*	MePR-9eR	CTCCATAGTAGGAGTAGTGTC	Irigoyen et al (2020)
5	*MeHSP90.9*	QMeHSP90.9F	CGCTATATATGCTCCGCAAA	Wei et al (2021)
6	*MeHSP90.9*	QMeHSP90.9R	TGAGATGAGAGATAAAAGGCACA	Wei et al (2021)
7	*MeSRS1*	QMeSRS1F	CCACCAACTGAAATCCCACA	Wei et al (2021)
8	*MeSRS1*	QMeSRS1R	CTCCTGCAACAAGTCCTACACC	Wei et al (2021)
9	*MeHsf8*	QMeHsf8F	TGAAGAAAATTCATAGACGGAAGC	Wei et al (2021)
10	*MeHsf8*	QMeHsf8R	CGTGGATATTTTCATTTTGCAC	Wei et al (2021)
11	*MeSYP121*	MeSYP121F	ATCCAGATGTCAGAGATGCC	Yoosomboon et al (2021)
12	*MeSYP121*	MeSYP121R	GAG CCTCCAAACGAACCTTA	Yoosomboon et al (2021)
13	*UBQ10*	MeUBQ_F	CGACTACAACATCCAGAAGG	Irigoyen et al (2020)
14	*UBQ10*	MeUBQ_Rev	TTGTGTCGGAACTCTCCACC	Irigoyen et al (2020)

Table S2 Dataset of Analysis of the expression of genes related to the accumulation of SA. The *PR-9e*, *PR-7f5*, *SPS1*, *SYP121*, *Hsf8*, and *HSP90* genes were detected in the C33, KU50, and Rayong11 genotypes.

Cultivars	DAI	$\Delta\Delta C_t$ Genes					
		PR-9e	SPS1	SYP121	PR-7f5	Hsf8	HSP90.0
C33	1	3.23	0.16	-0.68	2.88	-0.78	1.27
	2	-3.74	-3.24	-4.96	-2.09	-3.91	-1.47
	3	3.56	-8.57	1.89	3.87	1.54	-7.59
	4	14.03	15.62	11.32	13.79	9.66	13.25
	5	-0.75	-2.18	-3.94	-1.52	-5.27	-0.69
	6	0.84	-0.36	-1.71	1.11	-1.32	0.79
	7	-0.04	-0.35	-1.05	1.08	-1.75	0.33
KU50	1	-1.99	-1.45	-3.28	0.59	-1.16	1.80
	2	-1.94	-1.55	-2.38	0.46	-0.25	0.66
	3	-2.54	0.16	-5.39	-0.22	-2.74	0.00
	4	-4.92	-3.66	-5.29	-0.69	-3.82	-0.78
	5	-2.20	-0.16	-1.65	1.57	-1.67	2.12
	6	-3.27	-2.41	-3.81	-0.41	-1.56	0.06
	7	-3.02	-2.11	-3.83	-0.23	-1.69	1.24
R11	1	7.20	8.18	7.34	10.78	7.22	12.10
	2	7.87	8.94	7.20	10.89	9.36	10.60
	3	-4.20	-2.95	-4.29	-1.73	-2.65	-1.00
	4	-4.90	-3.05	-4.36	-2.06	-3.56	-1.08
	5	-3.27	-2.45	-3.91	-1.21	-2.81	-0.07
	6	-4.72	-3.56	-4.59	-0.64	-4.31	-2.43
	7	-3.65	-2.45	-4.04	-0.96	-1.96	0.24
